# Levofloxacin-containing triple therapy versus bismuth-based quadruple therapy as regimens for second line anti- Helicobacter pylori

**DOI:** 10.22088/cjim.10.2.211

**Published:** 2019

**Authors:** Mohamadreza Seyyedmajidi, Laleh Abbasi, Seyedali Seyedmajidi, Seyed Ashkan Hosseini, Anahita Ahmadi, Shahin Hajiebrahimi, Jamshid Vafaeimanesh

**Affiliations:** 1 Golestan Research Center of Gastroenterology and Hepatology-GRCGH (GOUMS), Golestan University of Medical Sciences, Gorgan, Iran; 2 Gastroenterology and Hepatology Diseases Research Center, Qom University of Medical Sciences, Qom, Iran; 3 Gastrointestinal and Liver Diseases Research Center, Iran University of Medical Sciences, Tehran, Iran

**Keywords:** *Helicobacter pylori*, Second-line Therapy, Levofloxacin-based triple therapy, Bismuth-based quadruple therapy

## Abstract

**Background:**

Although the prevalence of *Helicobacter pylori* infection decreased following the hygiene promotion and application of proper anti- H.pylori treatments, unfortunately gradual increase is reported in treatment failure; hence, application of a proper treatment regimen as a second-line therapy is of great importance.

**Methods:**

In the current randomized, clinical trial, a total of 120 patients with peptic ulcers who failed to respond to treatment were enrolled. In the OLA group, a regimen of omeprazole 40 mg/day, levofloxacin 1 g/day, and amoxicillin 2 g/ day was prescribed; however, a regimen of omeprazole 40 mg/day, bismuth sub-citrate 480 mg/day, furazolidone 400 mg/day, and amoxicillin 2 g/day was administered to the OFAB group. Both groups were treated for 2 weeks, and 6 weeks after the treatment, the urea breath test (UBT) was performed in the subjects. Collected data were analyzed with SPSS Version 18. At the end, 58 patients in group OLA and 57 patients in the OFAB group were analyzed.

**Results:**

According to the results of the current study, 96.7% of the subjects in the OLA and 95% in the OFAB groups completed the treatment course and the eradication rates were 86.7% and 78.3% in the OLA and OFAB groups, respectively (P=0.23). Treatment side effects were observed in 51.7% and 11.7% of the subjects in the OLA and OFAB groups, respectively (P<0.01).

**Conclusion:**

Both regimens were applicable as the second-line therapy due to insignificant difference between the results of the 2 groups; however, OLA regimen was superior to OFAB, due to lower side effects.

*Helicobacter pylori is a* Gram-negative bacterium and urease positive is the typical characteristic of the species, which can survive in gastric environment and successfully able to colonize the gastric antrum (1). Different studies indicated that *H. pylori* infection may be followed by different outcomes such as chronic gastritis, peptic ulcers, functional dyspepsia, and gastric cancer (2). In addition, multitude relationships were found between *H. pylori* infection and some extra intestinal diseases such as cardiovascular complications, diabetes, pulmonary involvements, hematological and neurological diseases, and glaucoma (3, 4). In a large study that covered 410 879 participants from 73 countries, among whom the overall prevalence of H. pylori was 44.3% (95%CI: 40.9-47.7) (5). In Iran, the prevalence of H. pylori infection has been reported to be at least 36% in Kurdistan and as high as 90% in Ardebil. (6). 

Different factors such as geographical properties, culture, and age, as well as socioeconomic factors influence the prevalence of *H. pylori* infection (8).Although the prevalence of infection is reduced by the hygiene promotion and application of proper anti-H. pylori treatments, unfortunately the gradual increase of treatment failure is simultaneously reported (9)to such an extent that the successful eradication rate of *H. pylori* dropped from 90% in 1990 to less than 60% currently (10). One of the main obstacles to the eradication of *H. pylori* is the antimicrobial resistance of these bacteria, particularly against clarithromycin probably caused by its indiscriminate administration for upper respiratory tract infections (11, 12). It is already confirmed that *H. pylori* can make efflux pumps over its cell wall for the rapid excretion of clarithromycin from bacterial cell and inhibit the attachment of clarithromycin to bacterial ribosome (12).The frequency of antibiotic resistance in *H. pylori* is different among the countries (13, 14). For example, increased resistance to clarithromycin and metronidazole in some Asian regions, such as Japan, reduced the potency of triple regimens to eradicate *H. pylori* (14). 

The treatment regimen should mainly focus on maintaining successful *H. pylori* eradication up to 85% and prevent the emergence of antibacterial resistance. The PPI (proton-pump inhibitors) triple therapy, including 2 antibiotics of clarithromycin + amoxicillin or metronidazole for 14 days is the most commonly administered regimen (14, 15). A meta-analysis compared the routine triple regimen with a bismuth-based quadruple regimen and reported the eradication of *H. pylori* in 80% of the patients who received the quadruple regimen and 79% of the ones who received the triple regimen, which indicated similar efficiency for the 2 regimens. Even it seems that the bismuth-based quadruple regimen was more useful for the cases who failed to respond to the routine triple regimen (16, 17).

 It seems that by the 20-year experience in the treatment of *H. pylori* infection, there is a long way ahead for researchers to find the ideal regimen. Different clinical trials as well as meta-analyses indicated that PPIs + 2 antibiotics were the commonest first-line therapy to eradicate *H. pylori*. This triple regimen fails to treat 20% of the patients and the rate even may be higher in adults. It seems that resistance to clarithromycin plays a significant role in the failure of treatment (18) regarding significant drug resistance and treatment failure in H. pylori infection, the study of various therapeutic regimens in the treatment of this infection has a special status. Nevertheless, the current study aimed at comparing 2 second-line therapies for the eradication of *H. pylori* in patients with peptic ulcers who previously failed to response the triple regimen as the first-line therapy.

## Methods

The current randomized controlled trial study was conducted on patients with peptic ulcer and failure to respond to the first-line therapy for *H. pylori* eradication who referred to Shahid Sayyad Shirazi Teaching Hospital in Gorgan, Iran. The study lasted from October 2016 to November 2017. After entering the study, each patient was given a number from 1 to 120. Each patient received a number (in a draw) for each number; there was a drug pack (the packages had the same shape) in the pharmacy. Half of the packets were OLA treatment regimen and the other half were OFAB-regimen. After the completion of the study, it was determined which drug was used by each patient. Then, a questionnaire including age, gender, medication, and clinical records was completed for each subject. 

**Figure 1:**
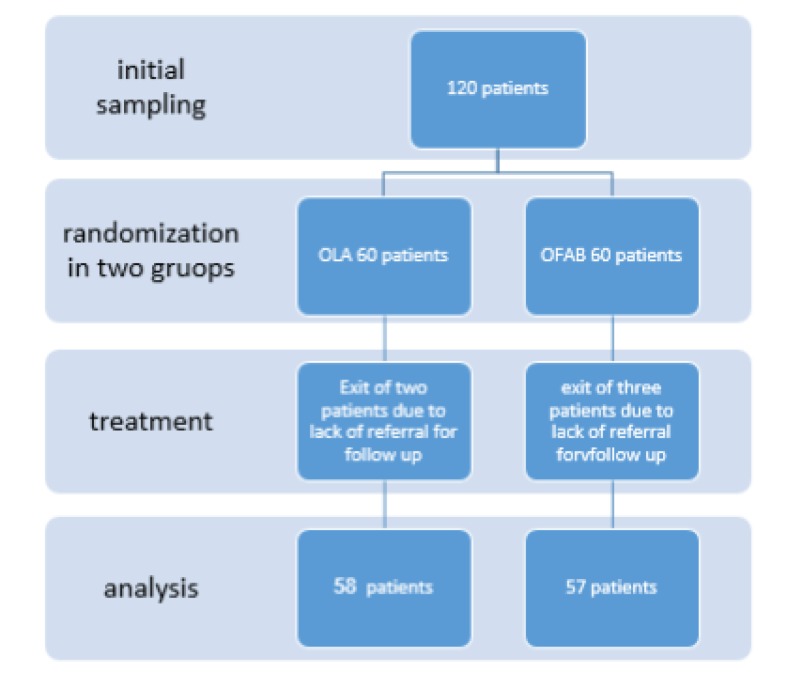
Consort flowchart (exclusion criterion was the irregular consumption of antibiotics due to severe side effects)

A total of 120 patients with peptic ulcer, caused by *H. pylori* infection*,* confirmed by histological examinations of biopsy specimens endoscopically taken from upper gastrointestinal (GI) tract with positive urea breath test (UBT) 6 weeks after completing a 2-week treatment course of a triple regimen including omeprazole 40 mg/day, clarithromycin 1 g/day, and amoxicillin 2 g/day, as the first-line therapy, were enrolled into the study. Subjects were allocated into 2 study groups using the random sampling method. (See fig 1: consort flowchart) In the OLA group, a second-line therapy for *H. pylori* eradication was administered to the subjects with a triple regimen including omeprazole 40 mg/day, levofloxacin 1 g/day, and amoxicillin 2 g/day for 2 weeks. In the OFAB group, also a second-line therapy for the eradication of *H. pylori* with a quadruple regimen was prescribed including omeprazole 40 mg/day, bismuth sub-citrate 480 mg/day, furazolidone 400 mg/day, and amoxicillin 2 g/day for 2 weeks. Six weeks after the completion of treatment course, UBT was performed and the level of eradication and treatment outcomes were compared between the groups. 

Collected data were analyzed based on the study objectives. The inclusion criteria were as follows: having peptic ulcer confirmed by endoscopy, receiving a complete course of first-line therapy, and resistance and failure to respond to the first-line therapy for H. pylori eradication; while the exclusion criterion was the irregular consumption of antibiotics due to severe side effects.

**Data analysis: **Collected data were codified and analyzed with SPSS Version 18. To express data, the frequency, percentage as well as mean and standard deviation (SD) were used. To compare age and gender distribution between the groups, the chi-square test and one-way analysis of variance (ANOVA) were used. To compare the eradication rate of *H. pylori* and results of UBT between the groups, chi-square test was conducted. Tables and figures were constructed to show the distribution of data. Also, p<0.05 was considered as the level of significance for all tests.

The protocol of the study was approved by the Research Ethics Committee of the local university and the required permission was given by the authorities of the hospital under study. The researcher introduced himself to the study subjects and explained the objectives of the study to them. Then, the written informed consent was signed by the study participants. The subjects were assured about the confidentiality of data at the end of the study. The participants were free to withdraw from the study at any stage. All practices and procedures performed in the current study had no mental or physical damage to the subjects and were designed based on the Code of Research Ethics developed in Golestan University of Medical Sciences, Gorgan, Iran. Due to the need for definitive drug therapy in the current study, drugs were provided by the patients, but the UBT was performed free of charge. Data were published anonymous. The research project was registered in the Iranian Registry of Clinical Trials (code no. IRCT2015123025770N1).

## Results

The mean±SD age of the study participants was 46.26±14.36 years; it was 45.7±14.7 years in the OLA and 46.8±14.1 years in the OFAB groups. No significant difference was observed between the groups regarding the mean age, based on ANOVA (P=0.68).

In addition, 68 subjects (56.66%) of the total population were males and 52 (43.34%) females. The groups were compared based on the gender distribution and results of chi-square test showed no significant differences between the groups in this regard (P = 0.71). In the current study, 58 (96.7%) subjects in the OLA group and 57 (95%) in the OFAB group completed the treatment course and the rest was eliminated due to poor medical compliance and lack of interest; there was also no significant difference between the groups regarding the number of drop outs.

The level of response to treatment was assessed in the groups and by the end of the study, totally 99 (82.5%) subjects responded to treatment. The analysis of frequency distribution in both groups showed positive response to treatment in 52 (86.7%) subjects in the OLA and 47 (78.3%) subjects in the OFAB groups. Results of chi-square test showed no significant difference between the groups regarding the rate of response to treatment (P=0.23).

Since the mean age of the study population was 46 years, the participants were allocated into 2 groups of >46 years as old age and <46 years as the middle age groups and the level of response to treatment was also assessed based on gender and age (table 1).

The level of response to treatment is shown in table 1 based on the age and gender and results showed no significant difference between the groups.

In addition, results indicated the incidence of side effects in 38 (31.7%) out of 120 subjects in the current study. For better explanation, drug side effects were observed in 31 (51.7%) subjects in OFAB and 7 (11.7%) subjects in OLA groups; results of the chi-square test showed a significant difference between the groups in this regard (p<0.01) (table 2).

**Table 1:** Medical data of the study groups

**Group**	**Response to Treatment**	**OLA** **% (N)**	**OFAB** **% (N)**	**Pvalue**
Male	Successful	90.9 (30)	82.9(29)	0.33
	Failed	9.8 (3)	17.1 (6)	
Female	Successful	81.5 (22)	72 (18)	0.42
	Failed	18.5 (5)	28 (5)	
<46 years	Successful	90.3 (28)	75 (21)	0.12
	Failed	9.7 (3)	25 (7)	
>46 years	Successful	82.8 (24)	81.2(26)	0.88
	Failed	17.2 (5)	18.8 (6)	

OLA: Omeprazole, Levofloxacin, Amoxicillin

OFAB: Omeprazole, Bismuth Sub-Citrate, Furazolidone, Amoxicillin

**Table 2:** Comparison of the level of side effects between the study groups

	**OLA**		**OFAB**		**P-value**
	**N**	**%**	**N**	**%**	
Nausea and vomiting	5	8.3	16	26.6	P<0.01
Abdominal pain	1	1.6	10	16.6	P<0.01
Diarrhea	0	0	4	6.6	P<0.01
Itching rashes	1	1.6	1	1.6	NS

NS: not significant

## Discussion

 In the current study, 58 (96.7%) subjects in the OLA group and 57 (95%) in the OFAB group completed the treatment course and the rest were eliminated due to poor medical compliance and lack of interest; there was also no significant difference between the groups regarding the number of drop outs. There is no exact ideal regimen as the first-line therapy for the eradication of *H. pylori*, and recommendation in this regard varies based on the antibiotic resistance pattern in each geographical region. For example, the resistance to clarithromycin varies from 18.9% in Asia to 29.3% in America and 11.1% in Europe (19). Recent reports have indicated the reduced efficiency of triple regimens on the eradication of *H. pylori* infection due to the increased resistance to clarithromycin and metronidazole caused by indiscriminate administration of these antibiotics in the Asian countries (20). A study in Iran reported the frequency to resistance to metronidazole and tetracycline as 55.6% and 38.1%, respectively; while, it was 7.3%, 7.3%, and 4.5% respectively for clarithromycin, amoxicillin, and furazolidone (21). In a study by Mokhtare et al., in Iran on 48 patients who failed to respond to a furazolidone-based regimen, a quadruple regimen including pantoprazole 40 mg/12 hours, amoxicillin 1 g/12 hours, bismuth sub-citrate 240 mg/12 hours, and clarithromycin 500 mg/12 hours was administered for 10 consecutive days and the subjects underwent UBT 8 weeks after the completion of treatment course and eradication of H. pylori was reported in 88.4% of the subjects (confidence interval (CI)95%: 74.91-96.11) (22). Recent clinical guidelines have recommended the application of bismuth-based quadruple regimens for 7 to 10 days as a second-line therapy to eradicate H. pylori (23). However, more recent studies have recommended increasing the treatment course to 14 days due to the significant differences between the results of 7- or 10-day and 14-day treatment courses (24).

 Valid recommendations on the treatment of H. pylori infection in patients failing first-line therapy are as below: After failure of PPI-clarithromycin-amoxicillin triple therapy, a bismuth-containing quadruple therapy or a fluoroquinolone-containing triple or quadruple therapy are recommended as a second-line treatment. And after the failure of a non-bismuth quadruple therapy, either a bismuth quadruple therapy or a fluoroquinolone-containing triple or quadruple therapy are recommended and after failure of bismuth-containing quadruple therapy, a fluoroquinolone-containing triple or quadruple therapy may be recommended. In cases of high quinolone resistance, the combination of bismuth with other antibiotics, or rifabutin, may be an option (25). The current study compared the effect of omeprazole, levofloxacin, amoxicillin with omeprazole, bismuth sub-citrate, furazolidone and amoxicillin 14-day regimen as the second-line therapy to eradicate *H. pylori*. The eradication rate was acceptable in both groups of the current study and the difference between the groups was insignificant (86.7% in OLA vs. 78.3% in OFAB).

Gu et al in China conducted a study on 138 patients 10 days after failure to respond to the treatment regimen. The patients received a 14-day quadruple regimen including pantoprazole, bismuth sub-citrate, and furazolidone plus ofloxacin or levofloxacin. The eradication level was 81.4% in the ofloxacin group based on the intention-to-treat analysis, while it was 87.7% in the per-protocol analysis, which both were significantly higher than those of the levofloxacin group (66.2% and 72.6%, respectively). They reported no side effects in the study groups (26). Xie et al., (26) in a study in China evaluated 720 patients with *H. pylori* infection and duodenal ulcer. The subjects were randomly allocated into each of the following groups:Groups 1 and 3 received rabeprazole 10 mg, amoxicillin 1000 mg, and furazolidone 100 mg twice a day for 7 and 10 days, respectively; groups 2 and 4 received rabeprazole 10 mg, bismuth sub-citrate 220 mg, amoxicillin 1000 mg, and furazolidone 100 mg twice a day for 7 and 10 days, respectively. The primary consequences of *H. pylori* eradication were assessed 4 weeks after the completion of the treatment course based on the intention-to-treat and per-protocol analyses, and the secondary consequences evolved the evaluation of changes in sign and symptoms 4 weeks after the completion of the treatment course. Drug side effects were reported in some of the subjects. Out of the total population, 666 patients completed the treatment course and were reexamined by UBT. The level of intention-to-treat in the study groups (1, 2, 3, and 4) was as follows 74.44%, 82.78%, 78.89%, and 86.11%, respectively. The rate of *H. pylori* eradication was significantly higher in group 4, compared with that of the group 1. Analysis of the results showed the *H. pylori* eradication ratein 4 groups respectively as 81.21%, 89.22%, 85.54%, and 92.26%. The side effects including dizziness, vomiting, diarrhea, nausea, rash, itching, and weakness and lethargy were self-limited (24). According to the results of the current study, there was a direct relationship between better response to treatment and longer treatment courses with more drug items.

Liang et al. (27) in China studied 61 patients with *H. pylori* infection who failed to respond a 7-day standard triple regimen. They were given a triple regimen including levofloxacin 500 mg/day, amoxicillin 1 g/12 hours, and esomeprazole 40 mg/12 hours and the eradication rate was reported 77% based on the intention-to-treat analysis and 81% based on the per-protocol analysis; their results were consistent with those of the current study. The rate of side effects and drug tolerance were 6.6% and 95.1%, respectively (28); the tolerance rate was similar to that of the current study. Accordingly, 96.7% of OLA group subjects and 95% of OFAB patients completed the treatment course. Although the rate of side effects was high in the current study and about half of the subjects in the OFAB and 11.7% in the OLA groups experienced side effects, most of the subjects completed the treatment course (28). The results of similar studies showed the increase of side effects following the application of furazolidone-based regimens; 51.7% of the subjects in OFAB group showed side effects, which is in agreement with findings about furazolidone. The limitation of this study is the need for large amounts of tablets to be taken by patients and also the need for referral patients to follow the eradication of H. pylori.

In conclusion, although no significant difference was observed between the groups regarding the *H. pylori* eradication rate, higher side efficiency of furazolidone-based regimens is a remarkable point in the current study. Hence, the application of furazolidone-free regimen as the second-line therapies is recommended in the current study; on the other hand, since Levofloxacin showed satisfactory results in the current as well as similar studies lower side effects of levofloxacin than furazolidone and bismuth suggests the involvement of this antibiotic in the *H. pylori *eradication regimens.
